# A Computational Framework to Identify Biomarkers for Glioma Recurrence and Potential Drugs Targeting Them

**DOI:** 10.3389/fgene.2021.832627

**Published:** 2022-01-17

**Authors:** Shuzhi Ma, Zhen Guo, Bo Wang, Min Yang, Xuelian Yuan, Binbin Ji, Yan Wu, Size Chen

**Affiliations:** ^1^ Department of Oncology, The First Affiliated Hospital of Guangdong Pharmaceutical University, Guangzhou, China; ^2^ Department of Pathology, Zhujiang Hospital, Southern Medical University, Guangzhou, China; ^3^ Academician Workstation, Changsha Medical University, Changsha, China; ^4^ Hunan Key Laboratory of the Research and Development of Novel Pharmaceutical Preparations, Changsha Medical University, Changsha, China; ^5^ Geneis (Beijing) Co., Ltd., Beijing, China; ^6^ Guangdong Provincial Engineering Research Center for Esophageal Cancer Precise Therapy, The First Affiliated Hospital of Guangdong Pharmaceutical University, Guangzhou, China; ^7^ Central Laboratory, The First Affiliated Hospital of Guangdong Pharmaceutical University, Guangzhou, China

**Keywords:** low grade gliomas, RNA-seq, differentially expressed genes, WGCNA, key driver genes, drug discovery

## Abstract

**Background:** Recurrence is still a major obstacle to the successful treatment of gliomas. Understanding the underlying mechanisms of recurrence may help for developing new drugs to combat gliomas recurrence. This study provides a strategy to discover new drugs for recurrent gliomas based on drug perturbation induced gene expression changes.

**Methods:** The RNA-seq data of 511 low grade gliomas primary tumor samples (LGG-P), 18 low grade gliomas recurrent tumor samples (LGG-R), 155 glioblastoma multiforme primary tumor samples (GBM-P), and 13 glioblastoma multiforme recurrent tumor samples (GBM-R) were downloaded from TCGA database. DESeq2, key driver analysis and weighted gene correlation network analysis (WGCNA) were conducted to identify differentially expressed genes (DEGs), key driver genes and coexpression networks between LGG-P *vs* LGG-R, GBM-P *vs* GBM-R pairs. Then, the CREEDS database was used to find potential drugs that could reverse the DEGs and key drivers.

**Results:** We identified 75 upregulated and 130 downregulated genes between LGG-P and LGG-R samples, which were mainly enriched in human papillomavirus (HPV) infection, PI3K-Akt signaling pathway, Wnt signaling pathway, and ECM-receptor interaction. A total of 262 key driver genes were obtained with frizzled class receptor 8 (*FZD8*), guanine nucleotide-binding protein subunit gamma-12 (*GNG12*), and G protein subunit β2 (*GNB2*) as the top hub genes. By screening the CREEDS database, we got 4 drugs (Paclitaxel, 6-benzyladenine, Erlotinib, Cidofovir) that could downregulate the expression of up-regulated genes and 5 drugs (Fenofibrate, Oxaliplatin, Bilirubin, Nutlins, Valproic acid) that could upregulate the expression of down-regulated genes. These drugs may have a potential in combating recurrence of gliomas.

**Conclusion:** We proposed a time-saving strategy based on drug perturbation induced gene expression changes to find new drugs that may have a potential to treat recurrent gliomas.

## Introduction

Gliomas are the most common type of central nervous system (CNS) tumors, which are composed of various distinct subtype tumors (Galbraith and Snuderl, 2021). Differed from non-CNS neoplasms, the gliomas’ grading system is quite complicate. The latest 2021 WHO Classification of Tumors of the Central Nervous System (WHO CNS5) has integrated certain molecular markers and histological features for more accurate staging of gliomas (Louis et al., 2021). Briefly, the gliomas can be classified as two categories, low-grade gliomas (grade 1–2) with a relatively benign slow-growing feature and favorable prognosis, and high-grade gliomas (grade 3–4) with highly infiltrative ability and malignant form. Glioblastoma multiforme (GBM) accounts for approximately 55% of gliomas and is considered as the most aggressive type of gliomas with a 5-years survival rate less than 5% and a median survival time of 12–15 months (Ostrom et al., 2015a; Ostrom et al., 2015b). Currently, the conventional therapeutic regimen for gliomas is surgical resection followed by radiotherapy and chemotherapy. However, the curative effect is far from satisfaction and recurrence is still the major obstacle to the success of chemoradiotherapy since the majority of GBM would experience recurrence within 6.2 months after diagnosis (Bähr et al., 2009; King and Benhabbour, 2021). Therefore, it is imperative to find new therapeutic target and novel therapeutic strategies for patient with gliomas.

Over the past decades, molecular biomarkers have gained important value in providing diagnostic information and therapeutic target for gliomas. Bevacizumab, an inhibitor of vascular endothelial growth factor (*VEGF*), was approved to treat recurrent GBM by the Food and Drug Administration (FDA) in March 2009 (Friedman et al., 2009; Kreisl et al., 2009). By targeting *VEGF*, Bevacizumab inhibits angiogenesis and blocks the nutrient supply, which ultimately impedes the growth and metastasis of GBM. In addition, methylation of the O^6^ -methylguanine-DNA methyltransferase (*MGMT*) promoter might serve as a predictive marker for temozolomide (TMZ) treatment response of GBM (Hegi et al., 2008). Poly (ADP ribose) polymerase (*PARP*) inhibitors can increase tumor sensitivity to TMZ chemotherapy and synergize with radiation therapy (Lesueur et al., 2018). A novel nano-compounds encapsulating wild-type p53 (SGT-53) could enhance the inhibitory effects of TMZ on TMZ-resistant GBM cells (Kim et al., 2015). Moreover, mutations/deregulation in the platelet-derived growth factor receptor alpha (*PDGFRα*), telomerase reverse transcriptase (*TERT*), epidermal growth factor receptor (*EGFR*), c-Myc, phosphatase and tensin homolog (*PTEN*), serine/threonine-protein kinase (*BRAF*) are frequently observed in glioma, which have become attractive markers for targeted therapy [Mukasa et al., 2010; Sampson et al., 2010; Killela et al., 2013; Johnson et al., 2014; Liu et al., 2020a; Liu et al., 2020b)].

Though a handful of molecular biomarkers have been discovered, the targeted therapies in clinical trials displayed limited curative effect for gliomas (Wu et al., 2021). This may be attributed to the inter- and intra-heterogeneity in driver mutations and plasticity of gliomas. The recurrent tumor might have a totally distinct gene expression signature in comparison with the primary tumor. Notably, 90% of druggable targets identified at initial diagnosis of gilomas are differentially expressed in a recurrent tumor (Schäfer et al., 2019). Ideally, a patient would select the specific drug according to his/her own molecular genetic feature and change the drugs over the course as the tumor evolves. With the advent of multiomics era, it is becoming possible.

In the era of big data, genome-wide molecular profiling at genome, transcriptome, proteome, and metabolome level have revealed comprehensive landscapes for all major types of gliomas. This has not only enriched our understanding of the molecular mechanism of gliomas pathogenesis and progression, but also broadened our ideas in discovering new therapeutic drugs. As we all know, new drug developing is a time-consuming course with high capital input and low yield, and drug repositioning based on computational tools could largely shorten the process (Liu et al., 2016; Xu et al., 2019; Zhou et al., 2019; Yang et al., 2020a; Liu et al., 2020; Tang et al., 2020; Zhou et al., 2020; Peng et al., 2021). In the present study, we proposed a fast, economical, and comprehensive strategy to find old drugs with new function for combating recurrence of gliomas. Our results on TCGA data suggested that this strategy provides a new direction in discovering drugs and brings hope for people in treating gliomas.

## Materials and Methods

### Data Collecting and Grouping

The RNA sequencing data of gliomas samples were downloaded from TCGA database. The samples were divided into 4 groups: low grade gliomas primary tumor samples (LGG-P, *n* = 511), low grade gliomas recurrent tumor samples (LGG-R, *n* = 18), GBM primary tumor samples (GBM-P, *n* = 155), and GBM recurrent tumor samples (GBM-R, *n* = 13).

### Screening of DEGs in Gliomas

The R package DESeq2 was applied to identify DEGs in the following data pairs: LGG-P *vs* LGG-R, GBM-P *vs* GBM-R. |log2fold change (FC)| ≥ 2, false discovery rate (FDR) < 0.5 and adjusted *p* value < 0.001 were set as threshold. R package clusterProfiler was used for Gene Ontology (GO) enrichment analysis and calculations. The enriched pathways in the up- or down-regulated gene set were generated using R package ggplot2.

### Weighted Gene Correlation Network Analysis and Key Driver Analysis

Network-based methods have been widely used to analyze the associations between various biological entities (Chen et al., 2018; Peng et al., 2018; Peng et al., 2020; Zhang et al., 2021). The R package WGCNA was used to construct a weighted gene co-expression network. The key driver analysis was performed using a software package described by Yang et al.(Yang et al., 2016). The first step was to generate a subnetwork NG, which is located within 2 steps of nodes in a given gene set. Next, the dynamic neighborhood search (DNS) was used to find the gene within 2 steps of each gene in NG. Lastly, by taking the gene set in the first step as the background, the hypergeometric test is carried out to calculate the enrichment value between the gene set in the second step and the input gene set. *p* value < 0.05 for DEG and *p* value < 0.01 for subnet were set as threshold in the key driver analysis.

### Drug Discovery

Based on the DEGs and key drivers, the CREEDS database was used to find potential drugs. The CREEDS database contains 906 drug perturbation gene expression signatures collected from GEO database (Wang et al., 2016). We screened the drug-gene pairs to identify potential drugs that could reverse the expression of DEGs and key drivers in gliomas. *p* value < 10–^10^ was set as the threshold.

## Results

### A Computational Framework to Identify Biomarkers for Glioma Recurrence and Potential Drugs Targeting Them

**FIGURE 1:**
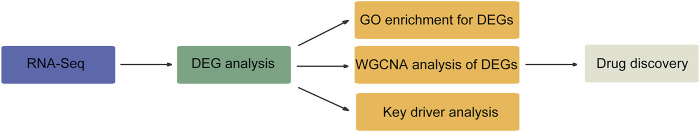
The framework of this study. The RNA-seq data was used to find DEGs, which was followed by GO enrichment, WGCNA, and key driver analysis. Potential drugs that could reverse the DEGs and key drivers were screened through the CREEDS database.

We proposed a computational framework biomarker identification and drug discovering for glioma recurrence **(** Figure 1 **)**. Firstly, the differentially expressed genes (DEGs) of primary gliomas samples and recurrent gliomas samples were identified from RNA sequencing data downloaded from The Cancer Genome Atlas (TCGA) database. Secondly, weighted gene correlation network (WGCNA) analysis and key driver analysis were conducted to find co-expression modules and key driver genes. Thirdly, the CREEDS database was applied to find potential drugs that could reverse the DEGs and key drivers. We then applied this framework to the downloaded TCGA data and identified important genes involving in glioma recurrence and potential drugs targeting them.

### Many DEGs Were Identified Between LGG-P and LGG-R, and Between GBM-P and GBM-R

**FIGURE 2:**
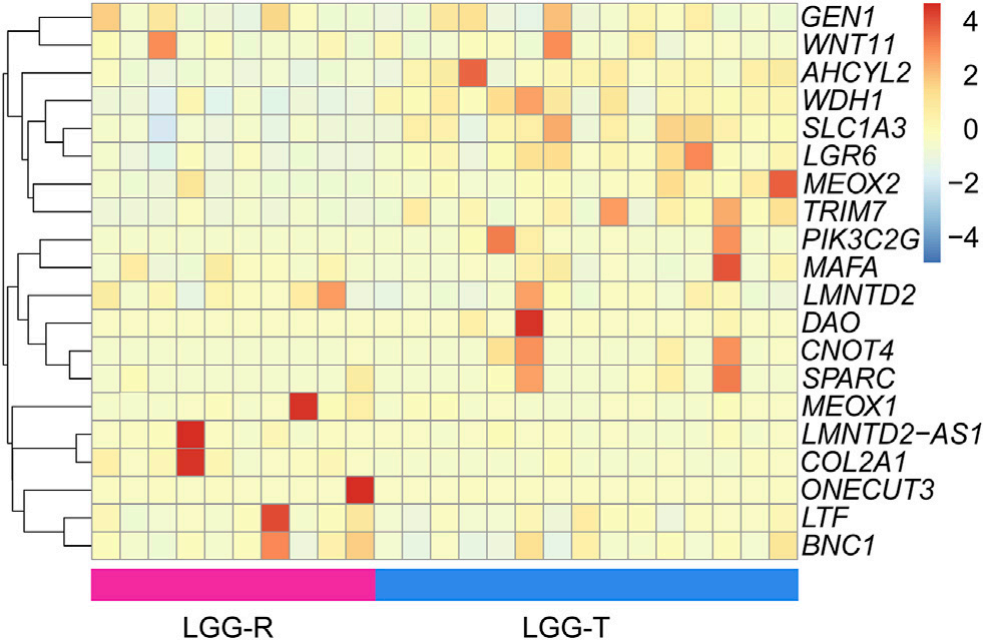
The heat map of the DEGs between LGG-P and LGG-R samples. Dao, mdh1, slc1a3, CNOT4, meox2, pik3c2g, LGR6, ahcyl2, SPARC and LTF were the top 10 differentially upregulated genes; LMNTD2-AS1, MAFA, COL2A1, ONECUT3, MEOX1, BNC1, LMNTD2, GEN1, Wnt11 and trim7 were the top 10 differentially down regulated genes.

We conducted a comprehensive analysis of the DEGs between LGG-P and LGG-R, and between GBM-P and GBM-R. The difference between GBM-P and GBM-R samples was not significant as we only obtained 2 upregulated and 29 downregulated genes. A total of 205 DEGs with 75 upregulated and 130 downregulated genes were identified between LGG-P and LGG-R samples. The specific details of each DEGs were shown in Supplementary Table S1. Since the number of DEGs between GBM-P and GBM-R was not large enough, we chose LGG-P and LGG-R pairs for further study. We randomly selected 25 samples to draw the heat map and the top 10 differentially expressed genes were shown in Figure 2.

**FIGURE 3:**
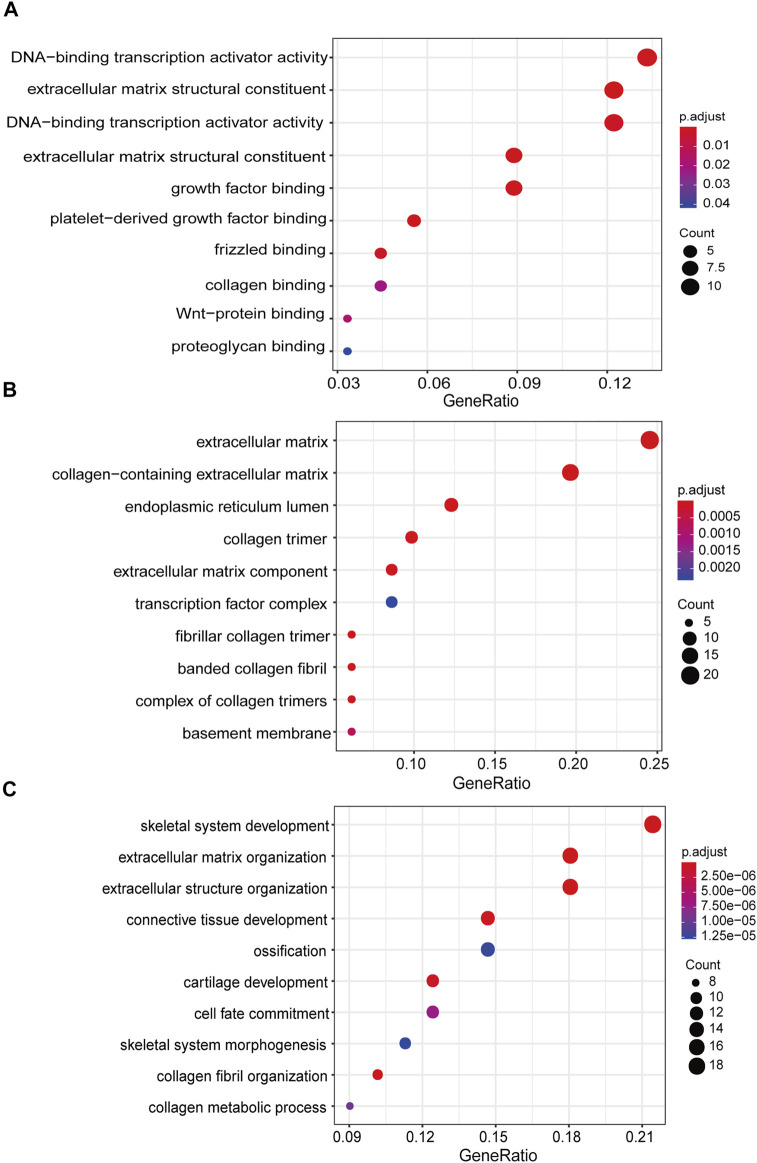
GO analysis of the downregulated genes between LGG-P and LGG-R. **(A)** molecular function category; **(B)** cellular component category; **(C)** biological process category. The *X*-axis is the ratio of differentially expressed genes enriched in the corresponding pathway, and the *Y*-axis is the name of the pathway.

**FIGURE 4:**
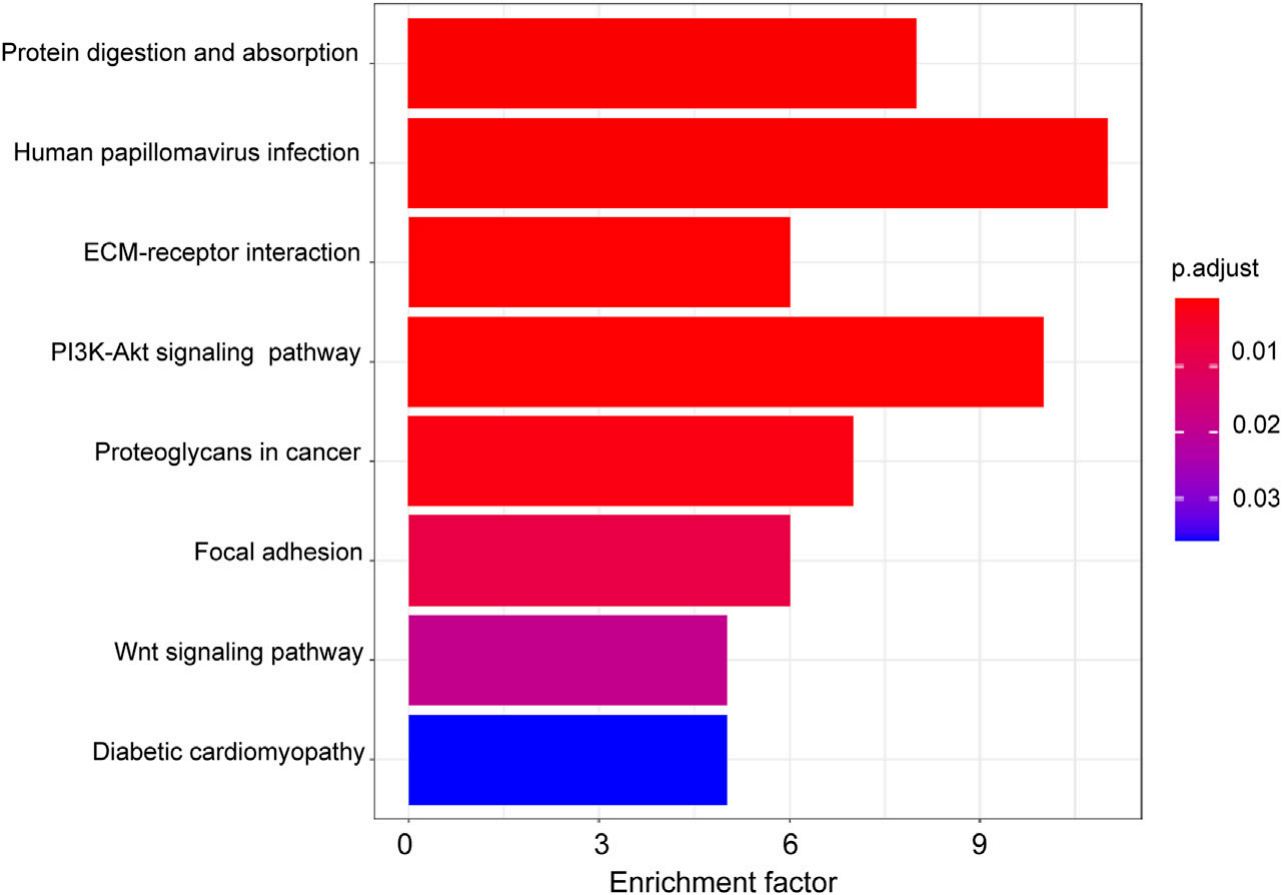
KEGG analysis of the downregulated genes between LGG-P and LGG-R. The DEGs were mainly enriched in protein digestion and absorption, human papillomavirus infection, ECM-receptor interaction, PI3K-Akt signaling pathway, and proteoglycans in cancer pathways.

GO analysis was utilized to annotate the function of DEGs between LGG-P and LGG-R. The upregulated genes could not be enriched owing to the relatively large *p* value. The downregulated genes were predominantly enriched in DNA-binding transcription activator activity, extracellular matrix structural constituent, growth factor binding, and platelet-derived growth factor binding for the molecular function (MF) category (Figure 3A). For the cellular component (CC) category, the downregulated genes were correlated with extracellular matrix, collagen-containing extracellular matrix, endoplasmic reticulum lumen, and collagen trimer (Figure 3B). For the biological process (BP) category, the downregulated genes were mainly involved in skeletal system development, extracellular matrix organization, extracellular structure organization, and connective tissue development (Figure 3C). KEGG enrichment analysis was further performed to explore the underling pathological pathways for LGG. As shown in Figure 4, the enrichment pathways include human papillomavirus infection, PI3K-Akt signaling pathway, Wnt signaling pathway, ECM-receptor interaction, and proteoglycans in cancer.

### Coexpression Analysis Revealed Chemical Synaptic Transmission Pathway and T Cell Activation Pathway as Key Modules for Glioma Recurrence

**FIGURE 5:**
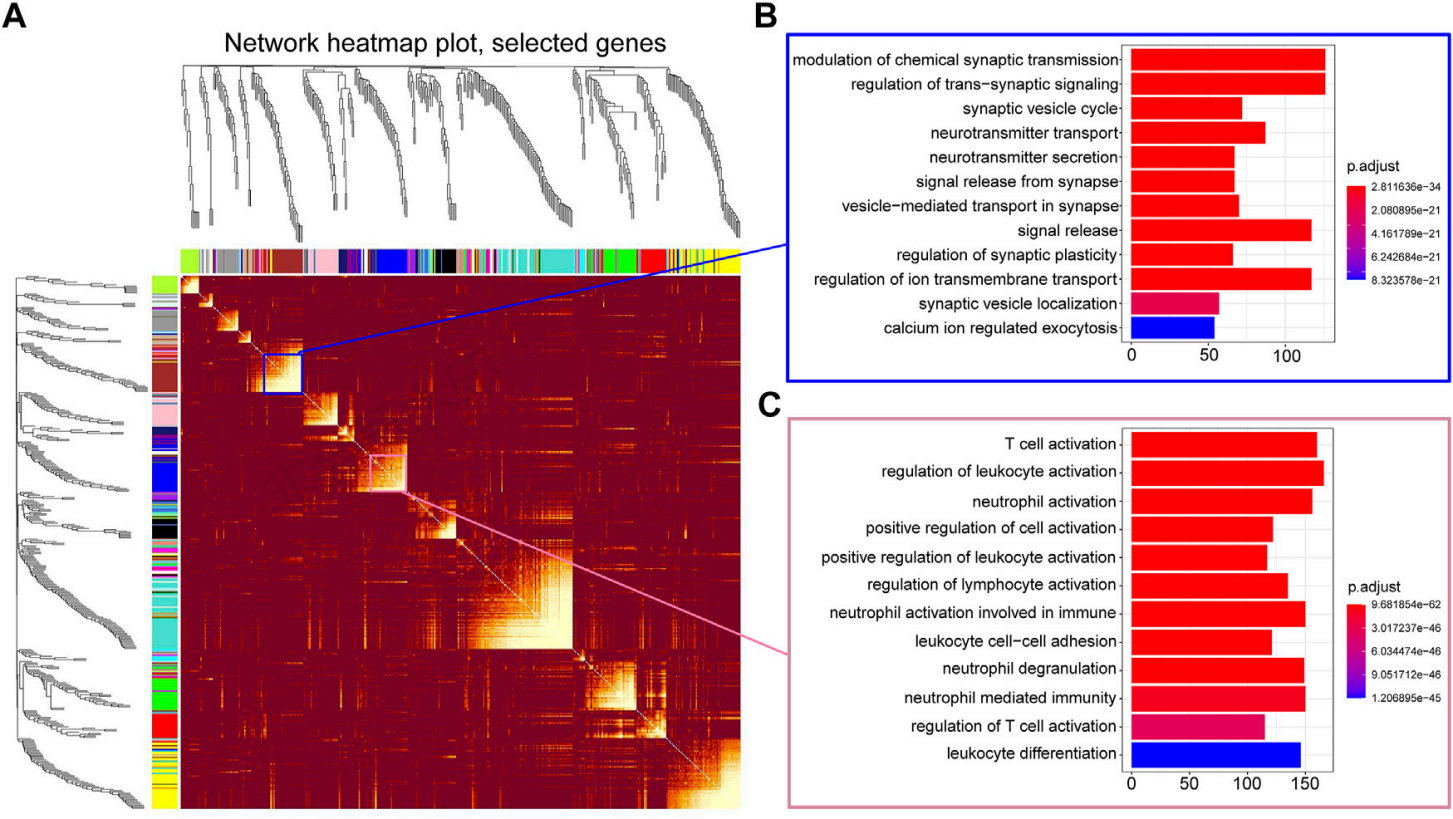
Highly correlated gene clusters were identified by WGCNA. **(A)** Topological overlapping heat map of 400 genes. **(B)** GO enrichment analysis of module 5. **(C)** GO enrichment analysis of module 8.

To better understand the function of differentially expressed genes, WGCNA was carried out to identify highly correlated gene clusters. Genes with zero expression were deleted from all samples, and the samples of some separated groups were removed from the hierarchical clustering results. WGCNA finally yielded 150 significant gene modules in LGG-P group and 65 gene modules in LGG-R group. Since there are too many genes to visualize, we randomly selected 400 genes to construct a topological overlapping heat map (Figure 5A) and performed functional enrichment analysis. As shown in Figure 5B and Figure 5C, the highly coexpression genes were mainly enriched in chemical synaptic transmission pathway and T cell activation pathway.

### Many Genes Including *FZD8* and *GNG12* Were Identified as Key Driver Genes for Glioma Recurrence

**FIGURE 6:**
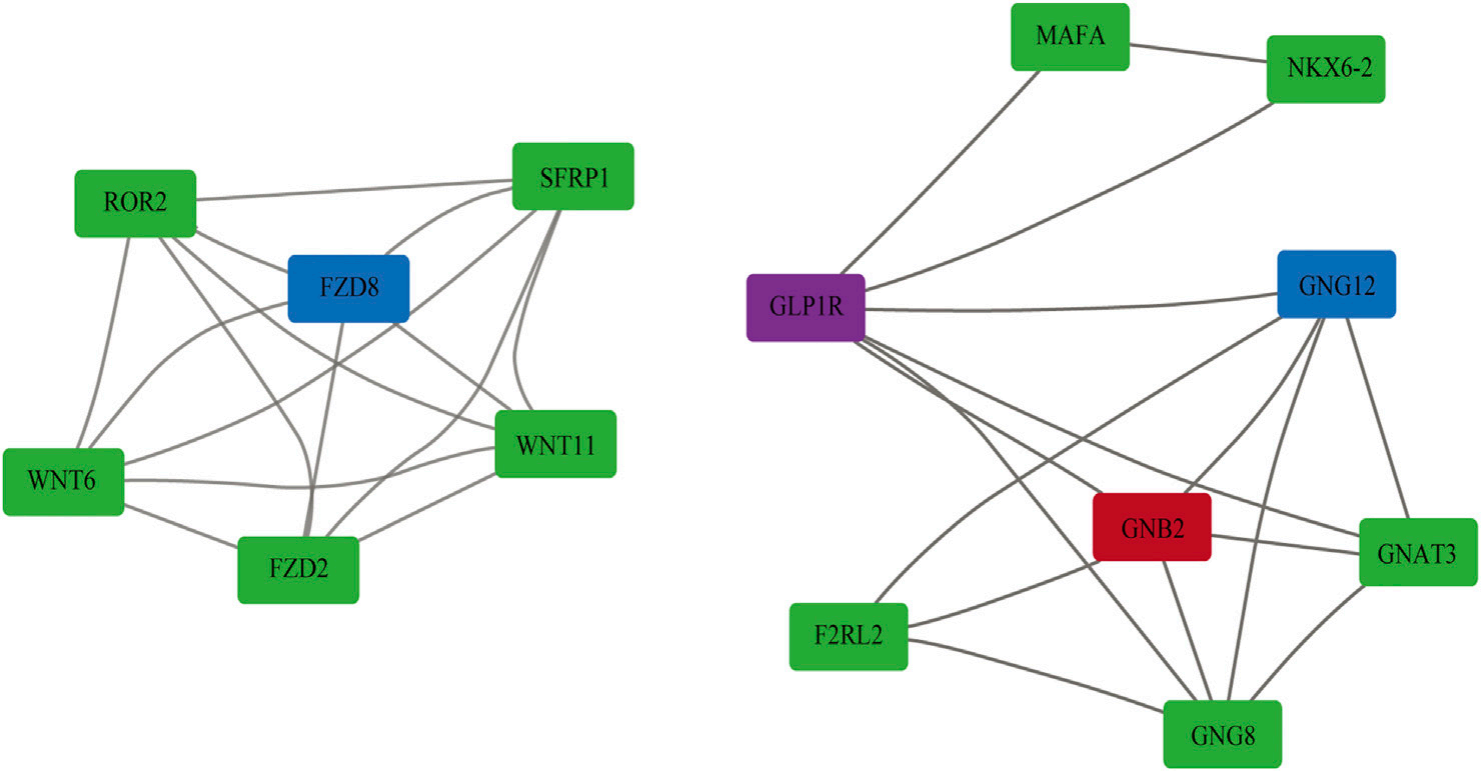
A subnetwork of the key drivers that connected the up and down regulated genes. Purple indicates upregulated genes, red indicates key drivers of upregulated genes, green indicates downregulated genes, and blue indicates key drivers of downregulated genes.

Key driver gene was considered as the hub gene that connected the up- or downregulated genes. For LGG-P *vs* LGG-R pair, we obtained 2 key drivers in the upregulated gene set and 260 key drivers in the downregulated gene set. The detailed information of the key drivers can be found in Supplementary Table S2. The most siginificant key drivers and their corresponding subnetwork were shown in Figure 6. We demonstrated that frizzled class receptor 8 (*FZD8*) and guanine nucleotide-binding protein subunit gamma-12 (*GNG12*) were two of the hub gene of the downregulated genes. *FZD8* is a G protein-coupled receptor protein that plays an important role in β-catenin signaling pathway and regulates cancer invasion and metastasis (Li et al., 2017). *GNG12* is a member of the G protein family and participate in a handful of trans-membrane signal transducer pathways (Yuan et al., 2021). G protein subunit β2 (*GNB2*) is a hub gene of up regulated genes and belongs to the guanine nucleotide-binding proteins family. *GNB2* may activate the canonical G protein signaling and involved in cancer initiation and progression (O’hayre et al., 2014). The functions and implications of these hub genes in cancers will be discussed further.

### A Few Drugs Including Paclitaxel and Fenofibrate Were Identified as Potential Drugs for Preventing Glioma Recurrence

Drugs that have a potential to reverse the expression of DEGs may be valuable for further treatment of gliomas. We obtained 26 drugs that could perturb the expression of up regulated gene sets and 50 drugs that could perturb the expression of down regulated gene sets by CREEDS database. In consideration of drug profile and previous studies, we focused on 4 perturbation drugs of up regulated genes (Paclitaxel, 6-benzyladenine, Erlotinib, Cidofovir) and 5 perturbation drugs of down regulated genes (Fenofibrate, Oxaliplatin, Bilirubin, Nutlins, Valproic acid) (Table 1). These drugs may provide new insight into preventing recurrence of LGG.

**TABLE 1:** Potential drugs for the treatment of LGG recurrence.

Class	Drug	Antitumor mechanism	Evidence (DIO)
up	Paclitaxel	prevents mitosis, blocks cell cycle progression, and inhibits cell growth.	10.1186/s11658-019-0164-y
up	6-benzyladenine	stimulates cell division, and inhibits respiratory kinase, leading to plant growth and development.	10.1111/plb.13154
up	Erlotinib	inhibits tyrosine kinase activity, blocks EGFR signaling pathway	10.1016/bs.podrm.2019.10.004
up	Cidofovir	inhibits viral DNA polymerase	10.1542/peds.2019-1632
down	Fenofibrate	activates PPARα-RXR signaling	10.7150/jca.24488
down	Oxaliplatin	blocks DNA replication	10.1016/j.ctrv.2020.102112
down	Bilirubin	an endogenous metabolite from haem	10.1021/np4005807
down	Nutlins	binds to p53/MDM2 complex and displace p53 protein	10.2174/138161210791033932
down	Valproic acid	inhibits histone deacetylase (HDAC)	10.2174/1574892810666150317144511

## Discussion

Gliomas are highly malignant tumors and recurrence is still the main obstacle to treatment. Though researchers have identified a handful of biomarkers for glioma, the therapeutic effect of targeted drugs is far from satisfaction. Currently, our study provides a fast, economical, and comprehensive method for finding potential drugs to treat gliomas.

Based on RNA sequencing data of LGG-P and LGG-R samples from TCGA, we yielded 75 upregulated and 130 downregulated genes, which were predominantly correlated with human papillomavirus (HPV) infection, PI3K-Akt signaling pathway, Wnt signaling pathway, ECM-receptor interaction. HPV infection is a major cause of cervical cancer, and associated with several epithelial malignancies, including oral cavity, anal, oropharyngeal, penile, vulvar, vaginal, and laryngeal cancers (Lu et al., 2020). Until now, there is no direct evidence indicates that HPV infection is involved in gliomas. We speculate HPV infection may interfere host immune system and participate in LGG recurrence. PI3K-Akt signaling pathway and Wnt signaling pathway are two of the canonical signaling transduction pathways in various cancers. PI3K/Akt pathway controls cell fate by regulating cell growth, apoptosis, angiogenesis, metabolism, autophagy, and chemotherapy resistance of gliomas (Shahcheraghi et al., 2020). Activation of PI3K-Akt pathway is associated with migration and invasion of glioblastoma cells (Huang et al., 2018). Wnt/beta-catenin signaling pathway plays a vital role in ionizing radiation-induced invasion of glioblastoma cells (Dong et al., 2015). It has been reported that high level of beta-catenin was associated with a poor prognosis in glioblastoma patients (Gao et al., 2017). Currently, a number of PI3K inhibitors and wnt inhibitors have entered clinical trials for gliomas treatment, such as BKM120, XL147 and XL765 (Lee et al., 2016; Zhao et al., 2017). ECM-receptor interaction pathway mediates cell migration by regulating neovascularization and diffuse infiltration of tumor cells (Cui et al., 2018). Previous study also indicated ECM-receptor interaction pathway was abnormal in development and survival of glioblastoma (Bo et al., 2015; Yang et al., 2020b).

We also identified several key driver genes that contributed to LGG recurrence, including *FZD8, GNG12, GNB2*. *FZD8* could activate the β-catenin pathway and play a vital role in cancer invasion and metastasis (Chen et al., 2020). Aberrant expression of *FZD8* has been reported in gastric cancer, prostate cancer, renal cell carcinoma, lung cancer, pancreatic adenocarcinoma, and overexpression of *FZD8* was considered to promote tumor metastasis (Li et al., 2017; Yang et al., 2017; Liu et al., 2019; Chen et al., 2020; Li et al., 2021). In addition, overexpression of *FZD8* leaded to chemotherapy resistance in breast cancer patients (Yin et al., 2013). *GNG12* acted as an important modulator or transducer in various transmembrane signaling systems. Researchers have demonstrated that *GNG12* could regulate cancer cell proliferation, inflammatory response, and immune response *via* activating the mTORC1 pathway and NF-κB signaling pathway (Larson et al., 2010; Luo et al., 2018; Li et al., 2020). *GNB2* was involved in cancer initiation and progression by activating AKT/mTOR pathway, MAPK pathway, and Hippo signaling pathway. Mutations of *GNB2* may result in targeted kinase inhibitors resistance to numerous types of cancer (Yoda et al., 2015). The roles of *FZD8, GNG12, GNB2* have not been fully illustrated in gliomas and needs further investigation. These key driver genes may help for understanding the pathogenesis of for LGG recurrence and shed new insight for developing new drugs.

However, developing new drugs from a molecular biomarker is a great project and still has a long way to go. In the present study, a total of 9 drugs with potential therapeutic effect against LGG recurrence were selected through a drug-gene perturbation method. Paclitaxel is a natural anticancer drug that has been widely used in the therapy of breast cancer, ovarian cancer, lung cancer, and several head and neck cancers. Paclitaxel binds to tubulin, promotes its assembly with microtubules and inhibits dissociation, which finally prevents mitosis and hinders cell cycle progression (Zhu and Chen, 2019). Erlotinib is used to treat some types of lung cancer and advanced or metastatic pancreatic cancer in clinical. Erlotinib blocks EGFR pathway by inhibiting tyrosine kinase activity and impeding cell proliferation, apoptosis, angiogenesis, invasions, and metastasis. Benzylaminopurine is a first generation cytokinin that stimulates cell division and inhibits respiratory kinase, leading to plant growth and development. Cidofovir is used to treat cytomegalovirus (CMV) infection through inhibiting viral DNA polymerase (Alcamo et al., 2020). Cidofovir also has a strong activity against herpes simplex virus (HSV), varicella zoster virus (VZV), adenovirus (AV), and human papillomavirus (HPV). Fenofibrate is widely used as a lipid-lowering drug through activating PPARα-RXR signal and transcription of lipid metabolism related genes. Numerous evidence has indicated that fenofibrate might exert anticancer effects through regulating cell apoptosis, cell-cycle arrest, invasion, and migration (Lian et al., 2018). Oxaliplatin is a third-generation platinum analog that has been widely used as the first-line drugs for metastatic colorectal cancer. It blocks DNA replication by binding to DNA and forming cross-linked DNA adducts, which consequently leading to cancer cell death (Mauri et al., 2020). Bilirubin is an endogenous metabolite from haem. Recent studies have indicated bilirubin levels may serve as biomarker for several cancers and vascular disease (Horsfall et al., 2020; Seyed Khoei et al., 2020). Nutlins is a small molecule that could displace p53 protein from p53/MDM2 complex, thereby preventing the degradation of p53. It has been revealed that Nutlins could induce p53 dependent cell cycle arrest and apoptosis in a number of tumors (Impicciatore et al., 2010). Valproic acid (VPA), a histone deacetylase (HDAC) inhibitor, is widely used to treat epilepsia, bipolar disorders, migraine, and schizophrenia. In addition, VPA may exert anti-tumor activity by regulating cell proliferation, apoptosis, differentiation, adhesion, invasion, migration, angiogenesis, and inflammation (Michaelis et al., 2007). As shown above, some candidate drugs were already commonly used in clinical, some candidates only showed preclinical antitumor activity. Weather these drugs/agents could prevent LGG recurrence still needs further preclinical and clinical trial validation.

In conclusion, we conducted a comprehensive analysis of LGG-P and LGG-R samples to find DEGs and key driver genes. By using a drug-gene perturbation method, a serious potential drugs/agents were screened to treat LGG recurrence. However, the exact effect of these drugs on glioma recurrence needs further experimental data for verification. Besides, independent dataset with paired primary tumor and recurrent tumor samples is needed to validate the findings in the future. This study may broaden our understanding of the molecular mechanism of LGG recurrence and provide new sights for drug discovery.

## Data Availability

The original contributions presented in the study are included in the article/Supplementary Material, further inquiries can be directed to the corresponding author.

## Supplementary Material

Supplementary Table S1 DEGs discovered in LGG-P and LGG-R samples.

Supplementary Table S2 Key drivers discovered in LGG-P and LGG-R samples.
